# Integrating the social sciences in epidemic preparedness and response: A strategic framework to strengthen capacities and improve Global Health security

**DOI:** 10.1186/s12992-020-00652-6

**Published:** 2020-12-30

**Authors:** Kevin Louis Bardosh, Daniel H. de Vries, Sharon Abramowitz, Adama Thorlie, Lianne Cremers, John Kinsman, Darryl Stellmach

**Affiliations:** 1 Center for One Health Research, School of Public Health, University of Washington, Box 357230, Seattle, WA 98195-7230 USA; 2 Amsterdam Institute for Global Health and Development, Paasheuvelweg 25, 1105 BP Amsterdam, the Netherlands; 3 Independent Consultant, Boston, USA; 4 Independent Consultant, Berlin, Germany; 5 Amsterdam University Medical Centre, Meibergdreef 9, 1105 AZ Amsterdam, the Netherlands; 6 Department of Epidemiology and Global Health, Umeå University, Umeå, Sweden; 7 Department of Public Health Sciences, Global Health (IHCAR), Karolinska Institutet, Stockholm, Sweden; 8 Sydney School of Public Health, Faculty of Medicine and Health, University of Sydney, Edward Ford Building (A27) Fisher Road, Sydney, NSW 2006 Australia

**Keywords:** Health emergencies, Epidemics, Preparedness, Global Health security, Infectious disease, Social science, Anthropology, Governance

## Abstract

**Background:**

The importance of integrating the social sciences in epidemic preparedness and response has become a common feature of infectious disease policy and practice debates. However to date, this integration remains inadequate, fragmented and under-funded, with limited reach and small initial investments. Based on data collected prior to the COVID-19 pandemic, in this paper we analysed the variety of knowledge, infrastructure and funding gaps that hinder the full integration of the social sciences in epidemics and present a strategic framework for addressing them.

**Methods:**

Senior social scientists with expertise in public health emergencies facilitated expert deliberations, and conducted 75 key informant interviews, a consultation with 20 expert social scientists from Africa, Asia and Europe, 2 focus groups and a literature review of 128 identified high-priority peer reviewed articles. We also analysed 56 interviews from the Ebola 100 project, collected just after the West African Ebola epidemic. Analysis was conducted on gaps and recommendations. These were inductively classified according to various themes during two group prioritization exercises. The project was conducted between February and May 2019. Findings from the report were used to inform strategic prioritization of global investments in social science capacities for health emergencies.

**Findings:**

Our analysis consolidated 12 knowledge and infrastructure gaps and 38 recommendations from an initial list of 600 gaps and 220 recommendations. In developing our framework, we clustered these into three areas: 1) Recommendations *to improve core social science response capacities*, including investments in: human resources within response agencies; the creation of social science data analysis capacities at field and global level; mechanisms for operationalizing knowledge; and a set of rapid deployment infrastructures; 2) Recommendations to *strengthen applied and basic social sciences*, including the need to: better define the social science agenda and core competencies; support innovative interdisciplinary science; make concerted investments in developing field ready tools and building the evidence-base; and develop codes of conduct; and 3) Recommendations for a *supportive social science ecosystem*, including: the essential foundational investments in institutional development; training and capacity building; awareness-raising activities with allied disciplines; and lastly, support for a community of practice.

**Interpretation:**

Comprehensively integrating social science into the epidemic preparedness and response architecture demands multifaceted investments on par with allied disciplines, such as epidemiology and virology. Building core capacities and competencies should occur at multiple levels, grounded in country-led capacity building. Social science should not be a parallel system, nor should it be “siloed” into risk communication and community engagement. Rather, it should be integrated across existing systems and networks, and deploy interdisciplinary knowledge “transversally” across all preparedness and response sectors and pillars. Future work should update this framework to account for the impact of the COVID-19 pandemic on the institutional landscape.

## Introduction

Infectious disease epidemics are increasingly seen as social and political events that require socio-political awareness, responses and solutions. Social scientists and humanities scholars have engaged with epidemics in a variety of ways, and in varying degrees, since the early twentieth century [1–3]. However most of this has been orientated towards an academic audience and based outside the actions, systems and networks that define public health, humanitarianism and biomedical responses to international and national epidemic outbreaks.

The recognition that health security threats require a broader range of expertise outside traditional biomedical and epidemiological disciplines has increased significantly with each passing epidemic: the HIV/AIDS pandemic in the 1990s; SARS in 2002–03; Ebola in 2014–16; Zika 2016–17 and now COVID-19 [4–9]. This has led to repeated calls, voiced in multiple reports and expert reviews, for “more social science involvement” [10]. These calls are increasingly leading to strategic investments in operational social science capacities for public health emergencies; for example, the Centres d’Analyses des Sciences Sociales (CASS), during the 2019 Ebola outbreaks in DRC, the establishment of a global epidemic social science network (SONAR-Global), the establishment of a GOARN-Research Social Science Working Group, and the establishment of technical advisory groups and departments at the World Health Organization [11–13].

However, critical institutional, cultural, and political gaps exist that prevent social science insights on issues ranging from (mis) trust, (mis/dis) information, the acceptability of laws and mandates, human behavior and social norms, stigma and discrimination, the impact of geopolitics, and the unintended consequences of interventions, as currently seen in the COVID-19 pandemic, from being mainstreamed into epidemic response [14–17]. This has also been widely discussed in other areas of global health, from HIV/AIDS [18], malaria [19], TB [20], neglected tropical diseases (NTDs) [21], health system strengthening [22] and the social determinants of health [23].

Our research with biomedical and public health practitioners and social scientists from a wide range of disciplines has demonstrated that there is global recognition that insights and methodologies should be given more weight and attention in decision-making; however, it has often remained unclear what reform would look like or which investments are required and by whom to mainstream social science capacity in epidemic preparedness and response.

The aim of this article is to comprehensively outline the barriers to building stronger core epidemic social science capacities and competencies, and to share recommendations proffered by experts in the field. We present the results of a study commissioned for the 2019 Funders’ Forum on Social Science Research for Infectious Disease, convened by the GloPID-R (Global Research Collaboration for Infectious Disease Preparedness) network (https://www.glopid-r.org) and designed by a team of social science, infectious disease and epidemic response experts. The primary target audience for the analysis included this group of funders, who commissioned us to identify needed areas of investment in global, regional, academic, and country capacities to improve social science integration in epidemic response [24, 25]. This article presents the results of our analysis, which occurred just prior to the COVID-19 pandemic in mid-2019.

**Fig. 1:**
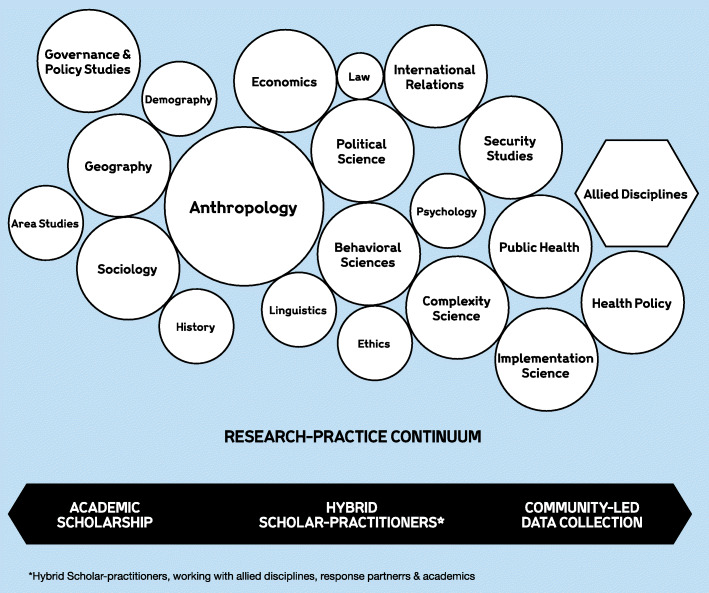
The disciplinary ecosystem

We repeatedly use the terms “social science” or “epidemic social science” as a non-denominational proxy for systematic, technical, operational research-based interventions. We recognize that this includes a wide array of areas of expertise (economics, psychology, sociology, anthropology, political science, development practice, etc) across a continuum, from academic scholarship to hybrid scholar-practitioners to community-led data collection (see Fig. 1). While medical anthropology, the behavioural sciences, and risk communication may have driven much of the recent (pre-COVID-19) momentum for structural change in how we do epidemic response, many other fields (ethics, international relations, history, etc) have also played important roles and the nature of future multi-disciplinary work is rapidly evolving [26, 27].

We define the role of social science in this field broadly to mean a holistic engagement with social, cultural, historical, economic and political factors as they affect, and are affected by, disease outbreaks, epidemics and pandemics, with a particular focus on the way people (individuals, families, communities, social networks, healthcare workers, local government, humanitarian responders and others) experience, engage and negotiate their circumstances. In this sense, we view social science as a means to support the humanization of epidemic response and generate a strong ethical alliance with local populations and health systems. It also challenges the tendency for epidemics to be viewed through an exclusively biomedical gaze with a focus on technical fixes, rather than as complex sociopolitical emergencies that require social, cultural, economic, political and health system solutions [28–32]. In this way, we see social science providing a critical, self-reflective lens to the practices public health actors often take for granted.

**Fig. 2:**
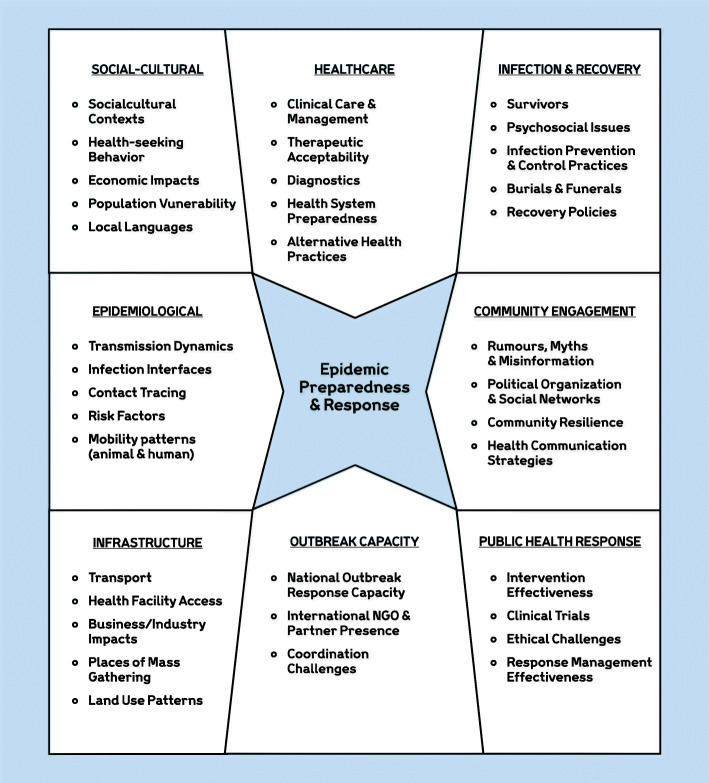
Informational Contribution of Social Science to Epidemic Response (The research team developed this figure by reviewing the various publications in the literature review and using our past experiences working in epidemic preparedness and response. It is not meant to be an exhaustive or conclusive list, but merely serves to illustrate the many different areas of engagement.)

Social science, like the diverse areas of study that comprise “public health and epidemiology,” can be understood as an “expert service” to support the traditional core pillars of epidemic response through knowledge-generation and analysis to support programming, policy, and research. Social sciences’ most notably services include assistance to community engagement (CE), social and behavioral change communication (SBCC), risk communication (RC), social mobilization and psychosocial support, but it increasingly is being leveraged to address clinical, vaccine research and distribution, security, social protection, and other areas across national public health, humanitarian cluster, and international systems and partnerships for coordinated alert and responses. In Fig. 2, we delineate some of the many topical areas where social science can contribute valuable insights.

Despite high expectations and interest, investments in integrating social science into epidemic response have not, as a rule, followed nearly the same proportion and scale as scientific disciplines such as epidemiology, disease modelling or virology. Although there is no formal analysis of financial flows, one estimate placed anthropological investments during the West African Ebola epidemic at less than 0.03% of the overall $10 billion response [26]. According to Larkan et al. [33], only 3% of WHO non-support staff have social science and legal skills and training required for epidemic preparedness. That said, there have been a few noteworthy advances from the situation just a few years ago, when efforts were more ad hoc and fragmented – See Table 1. The society-wide impact of the COVID-19 pandemic globally has further moved social science to the foreground. As of September 2020, the total amount of money allocated to social science research projects equals roughly 13% of total global COVID-19 research funding [34]. In addition, a COVID-19 Research Roadmap Social Science Working Group was established by the WHO in February 2020 [35]. These reflect growing awareness of the importance and contribution of social science.

**Table 1:** New projects and initiatives in epidemic social science

Key gains have been made in the following areas (documented prior to the COVID-19 pandemic):	
• There is a more clearly defined space for social science in the global epidemic response architecture in key organizations, including GOARN, WHO, UNICEF, OFDA, CDCs, IFRC, MSF and others. For example, social science has been prioritized as a key action area in the WHO, IFRC and UNICEF-lead COVID-19 Collective Service to Coordinate Risk Communication and Community Engagement (RCCE) for the global COVID-19 response.	
• UNICEF and partners has established the Centres d’Analyses des Sciences Sociales (CASS) model, during the 2019 Ebola outbreaks in DRC [12]. Through the CASS mechanism, UNICEF is supporting Ministries of Health in taking leadership on using applied social science for real-time public health responses. See: https://extranet.who.int/goarn/cass-social-science-support-covid-19-lessons-learned-brief-1	
• Major investments from the European Commission have been made to build social science capacity and networks, resulting in the Horizon 2020 Sonar-Global network [13]. See: https://www.sonar-global.eu	
• Canada’s Institute of Health Research (CIHR) has launched a global governance of infectious disease network initiative with a core focus on social science.	
• WHO tested its first social science for epidemics “boot camp” (SocialNet) in 2017 and a second version was conducted in Eastern Europe in 2018.	
• New clinical research networks (ALERRT (https://www.alerrt.global), Pandora-ID-Net (https://www.pandora-id.net), PREPARE (https://www.prepare-europe.eu/Networks)) have included social science components.	
• The Social Science and Humanitarian Action Platform (SSHAP) has developed and mobilized rapid synthesized knowledge briefs. See: https://www.socialscienceinaction.org.	
• A GOARN Social Science Research network has been established to coordinate research efforts during specific epidemic outbreaks. WHO has since established a COVID-19 Social Science Working Group, which has been integrating social science into its R&D Blueprint processes. See: https://www.who.int/publications/m/item/covid-19-social-science-working-group	
• WHO Joint External Evaluations have expanded the role of risk communication and community engagement assessments.	
• Social scientists are starting to be integrated into rapid support teams, most notably in the UK-Public Health Rapid Support Team (UK-PHRST) and the U.S. Centers for Disease Control.	
• Epidemiological Field Training Programs have begun including basic introduction to social science, and there is a growing level of interest to mainstream these trainings through the Africa CDC.	

Our initial aim was to draft a roadmap for global health funders, multilateral agencies, governments, public health institutes and universities; but in this paper, we seek to engage the wider scientific, medical, research, and practitioner communities in these ongoing discussions about how to improve public health emergency preparedness and response. It will take all hands on deck to transform these recommendations into a global framework, roadmap, and ultimately, a strategy.

The paper is divided into three sections (areas in our framework) that consolidate 38 recommendations, based on our analysis of an extensive number of interviews and other data. The first section discusses recommendations to 1) *improve core social science response capacities*, including human resources, data analysis capacities, mechanisms for operationalizing knowledge and rapid deployment. The second section discusses recommendations to 2) *strengthen applied and basic social sciences*, including the social science agenda, interdisciplinary science and field ready tools. The third section discusses recommendations for 3) a *supportive social science ecosystem*, including essential institutional development, training and capacity building. The discussion and conclusion reflect on the implications of our analysis.

## Methods

This research is grounded in a qualitative study design commissioned for the 2019 Funders’ Forum on Social Science Research for Infectious Disease, convened by the GloPID-R (Global Research Collaboration for Infectious Disease Preparedness) network (https://www.glopid-r.org). The study was conducted by senior social scientists with direct experience in epidemic preparedness and response; members of this team have worked in various capacities at the interface of epidemic social science, including with Ebola and Zika, and in collaboration with international agencies, INGOs, Ministries of Health, and academic groups. The study was part of a recommendations and prioritization exercise that has since informed global investments in social science capacity in both health and humanitarian emergencies. This article presents the results of our analysis, which occurred just prior to the COVID-19 pandemic in mid-2019.

This qualitative study involved four data collection activities carried out between February and April 2019. We first conducted a consultation workshop with 20 senior social scientists from Africa, Asia and Europe at the Horizon 2020 SoNAR-global consortium in 2019 (https://www.sonar-global.eu). This workshop solicited feedback on knowledge and infrastructure gaps, lessons from other disciplines, and funding priorities and recommendations. From the feedback generated during this workshop, we developed an initial list of codes that were used to structure a literature review of 128 peer-reviewed articles and technical reports.

We invited 105 key stakeholders from the social science, epidemiology, biomedical, global health and humanitarian fields to participate in remote interview consultations. In total, *n* = 75 interviews (30–60 min each) were completed. A question guide was developed for these open-ended interviews that included main and probing questions. Main questions focused on the experience of the key informant with social science and epidemic response, their opinion of the state of the field and recent progress, knowledge gaps, infrastructure gaps, conceptual issues about what we mean by “social science” and, finally, recommendations for funders. A breakdown of the institutional affiliation of these stakeholders is provided in Table 2. There was an over-representation of respondents associated with international organizations in the global north; but approximately 25% (*n* = 19) included respondents in the global south: Brazil (1), Pakistan (1), Sudan (2), Palestine (1), Nigeria (1), Myanmar (1), South Africa (1), Tanzania (2), Ethiopia (3), Liberia (1), Ukraine (1) and Kenya (4). We deliberately sought out key informants with long-standing and varied experiences and background in the field of epidemic response. This was done by asking Wellcome Trust to provide an initial list of key informants, which our team then purposively added to in order to select a balance of experienced informants from different organizations, scientific disciplines, and geographical locations.

**Table 2:** Institutional affiliation of *n* = 75 key informants

Institutional affiliation	Key informants
Social scientist based at a university	20
Non-social scientist based at a university	11
International NGO	13
UN agency	11
National health agency (CDC, MoH)	8
Think tank; research NGO	7
Funder	4
Public-private partnership	1
Total	75

Focus group discussions were also conducted with (1) members of the GOARN Research Social Science group, and (2) a team of social scientists from South Asia. We supplemented these primary responses with a review of 56 interview transcripts from the Ebola 100 project archive (https://ebola100project.net) with researchers and responders from the West African Ebola epidemic about the use and perception of social science data in the West Africa response.

Informed consent was obtained from all respondents. Ethical approval for the study was obtained through the University of Amsterdam Institute for Social Science Research (2019-AISSR-9918).

Analysis of this data was conducted in Microsoft Excel, which allowed for the rapid coding of knowledge and infrastructure gaps and linked them to causal factors and recommendations for priority investments. A coding framework was developed based on inductive thematic analysis of the initial consultation and literature review. This identified 28 themes, which were used to analyse the subsequent key informant interviews and focus group discussions. In our Excel analysis template we separated knowledge and infrastructure gaps and included the following columns: theme, gap, why it exists and recommendation to address it. We assigned unique anonymous codes to each interviewer. An initial list of 600 gaps and 220 recommendations emerged from the consultation, interviews, focus group and literature review data. The list of gaps was synthesized into 12 categories. The 220 unique recommendations were then ranked by the team of authors using a prioritization matrix. We ranked recommendations by valuation, urgency and expected impact using a numerical ranking scale of 1–3 (low, moderate, high). This ranking considered the number of times the recommendations was mentioned, by whom and how it fit with the identified list of gaps as well as the team’s prior knowledge and experience in this field. The team reviewed and ranked all 220 recommendations during two remote meetings. Through further analysis and synthesis, we developed a final list of 38 recommendations. We organized all of these results into three overarching areas, presented in the Results section.

Our analysis aimed to ground gaps and recommendations in the viewpoints and experiences of our study participants. Our primary aim was not to focus heavily on divergences of opinion but to outline and synthesize the diverse opinions and perspectives of different stakeholders in order to create a framework that could be used by funders to guide funding calls and areas of investments. Hence, some stakeholders certainly emphasized and highlighted some gaps and recommendations and not others. Our inductive analysis, however, did find a large degree of consensus on many of the most important issues. As noted below, a few of the recommendations include prioritization exercises that should be organized by different stakeholders to define which recommendations are most relevant from their organisational standpoint. Direct quotes from study participants, presented in the text below, were chosen due to their representativeness of a consensus position and not as an outlier position. When we refer to “respondents” we mean that this was a consensus viewpoint expressed by multiple respondents across different institutional affiliations.

Two limitations to our approach include: 1) the fact that the expert group’s composition consisted largely of scholars based in the global north; and 2) our initial plan to ask for feedback from a sample of our key informants on our analysis was not possible due to time limitations.

## Results

**Fig. 3:**
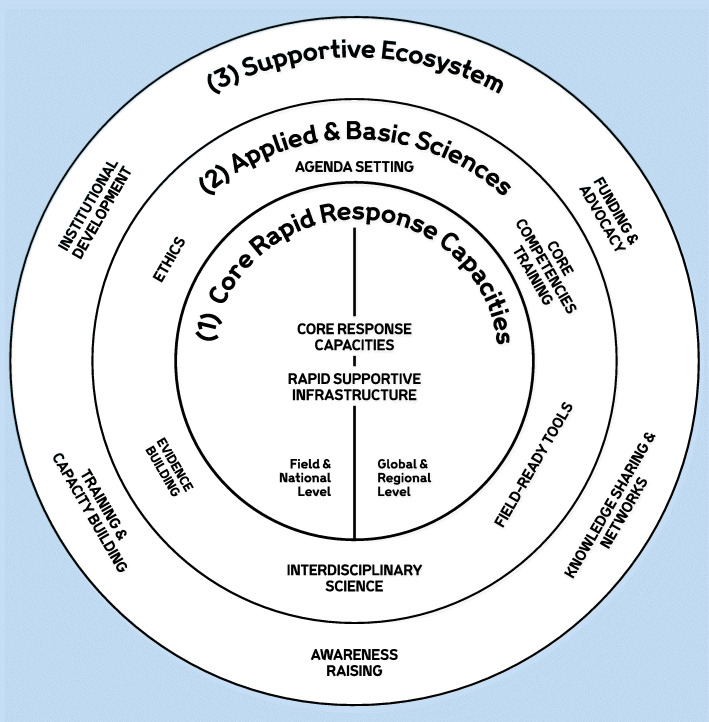
Strengthening Epidemic Social Science: Priority Areas for Building Global Capacity

In this section, we present our results according to three domains – 1) core response capacities; 2) applied and basic social science investments; 3) and the supportive ecosystem (Fig. 3) – and review findings for each subdomain. These are not mutually exclusive areas – they build upon each other and should all be considered essential areas for investment and engagement.

### Developing core rapid response capacities

**Fig. 4:**
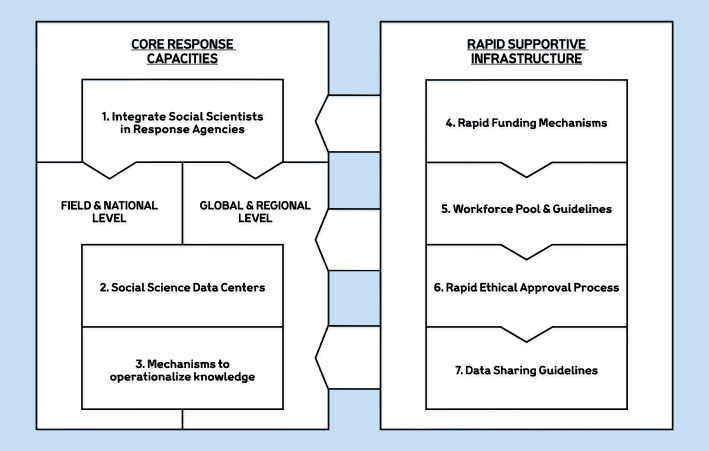
Core Capacities and Epidemic Infrastructure: Areas for Social Science Integration

There was a strong consensus among our study participants, from varying backgrounds and positions, that integrating social science was an important priority for epidemic response stakeholders and should begin with a set of core social science response capacities across the epidemic response ecosystem. Social scientists need to be able to provide critical insights and gain structured authority in the field and in policy roles. Our analyses organized a set of 7 recommendations, that emerged from our data, along two sub-domains: 1) core response capacities and 2) rapid supportive infrastructure (Fig. 4).

#### Core social science capacities for rapid response

*“Social scientists are often missing from senior operational discussions on preparedness and response” (Key informant)*

*“The problem is not that we’re not doing good social science. The problem is that the good social science is not finding its way into practice.” (Key informant*)

Respondents, from all different institutional affiliations, emphasized that the institutionalization of social science should begin with basic investments in human resources in existing response organizations and key public health institutes, and should occur simultaneously at global and national level with a focus on high-priority countries. UN agencies, like WHO and UNICEF, and others have relied on one (or fewer) focal persons to coordinate social science work, and their time and effort is partitioned between risk communication, community engagement, and social science. Coordination at national and field-level during epidemics and outbreaks is particularly problematic, due to the lack of a clearly defined coordinator to liaison between partners, teams and data streams. At the time of research, there were no permanent or temporary staffs in most organizations dedicated to integrating social science in epidemic preparedness and response. Reliance on short-term consultants may have impeded the growth of core capacities.

This lack of capacity and coordination means that integration occurs in a very piecemeal, informal fashion, without systematic networks available to orientate better quality social science fieldwork and engagement. Studies are done in isolation, on different timelines, and they are asking slightly different questions, generating non-aggregated data. There is also a clear need for agreed-upon mechanisms to feed social science information, insights, data, and analysis into decision-making at field and global levels. Social science data, and outcome data generally, are missing from Situation Reports. As one informant noted:

*We need a coordination mechanism for all of the social science data in a response. We need local data analysis for real-time feedback and we need aggregated data, to be analyzed over time to track trends. We need mechanisms to share and disseminate data that is constructive for the response. Social science data can be highly political and sensitive. (Key informant, UN agency)*

Many respondents indicated that the GOARN and GOARN research networks (or similar coordination-like networks) should play important roles in supporting integration and growth. Mechanisms or agreements for data sharing and for expert input into the design of research studies are lacking [24, 25].

Our analysis identified three strategies: 1) integrate social scientists into response organizations at multiple levels of the epidemic response architecture (which may require start-up grants to the major response organizations for core staff and activities in the early years); 2) create social science data centres at field and global levels to coordinate and integrate data across the pillar system (including integrating social science data with epidemiological and geospatial information; institutionalizing rapid contextual brief capacities; and including social science in Situational Reports); and 3) strengthen mechanisms that operationalize knowledge to influence decision-making, through developing SOPs, socialization of different pillars, formalization and mainstreaming activities.

#### Rapid support infrastructure

*“We should have a cluster of people, in every high priority country, trained in rapid social science field assessments, that can be called up in case of an outbreak.” (Key informant, social scientist)*

Infrastructure and knowledge architectures optimized for social science inputs will enable rapid deployment and mobilization. Respondents stated that many aspects of social science infrastructure (human, financial, and material resource mobilization) are not adapted for epidemic response. Secondment mechanisms to transfer social scientists into active epidemic situations are hampered by administrative and institutional barriers. This includes research ethics approvals. As one informant noted:

*“We could have deployed right away for the surveys but we had to wait two months for amendments from the ethics review board, even though we already had ethical approval and asked for expedited request! It was such an unnecessary delay.” (Key informant, social scientist)*

Donor efforts have been made to create “rapid funding mechanisms” for applied research in the medical humanitarian sector; for example, through Elrha in the UK (see: https://www.elrha.org). Fieldwork through these mechanisms is funded through mechanisms that review, grant, and disburse slowly, and social scientists arrive in the field well into the cycle of the epidemic. Funding disbursement mechanisms demand that contracts and conditions need to be in place before emergencies. One of our informants remarked:

*“Rapid research funds still take so long to get, the epidemic is over by the time it comes. This can lead to our research partners being forced to out loans in-country to start the work fast. This creates tensions … ” (Key informant, social scientist)*

Organizations that frequently respond to epidemics struggle to identify social scientists with the right experience, language skills, and expertise, and have difficulty identifying regional and country experts. In particular, emergency response work clashes with the pace, incentives and demand of university teaching and administrative responsibilities, where many social scientists are based.

Recommendations from our respondents focused on further institutionalization of rapid funding mechanisms for research during epidemics, so that teams could start research immediately at the start of an emergency. Support for a workforce pool and guidelines for deployment was also frequently mentioned, including the maintenance of a register by individual institutions and/or central coordinating agencies (e.g. WHO) of social scientists situated at the national level and a surge capacity roster of international experts. Lastly, we identified a particular gap in the lack of guidelines for data sharing and rapid ethical approval mechanisms; and hence a need for pre-approval and socialization of ethical protocols, particularly in high-priority countries, as well as the support for the development and approval of data sharing guidelines and agreements by major response agencies.

### Strengthening social science across the applied-academic continuum

**Fig. 5:**
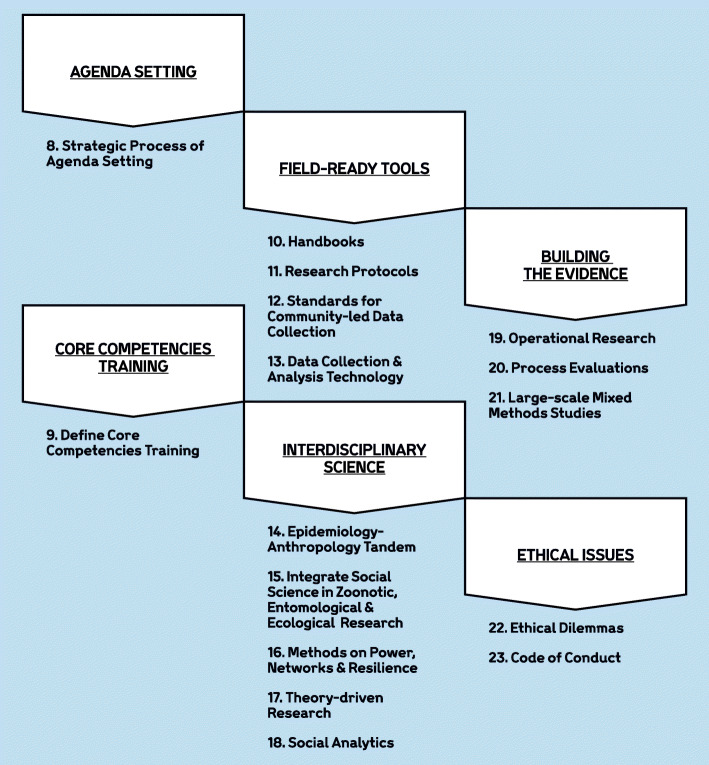
Priorities for strengthening basic and applied epidemic social science

Our analysis identified a second area of capacity strengthening: applied and basic social science. Investments in this area would strengthen core rapid response capacities (Area 1) by supporting the operationalization and optimal interdisciplinary growth and innovation of the field. Our analysis showed that respondents believed that this should occur simultaneously across the spectrum of applied and scholarly science (albeit participants based at response agencies were more concerned with the former, while social scientists emphasized the importance of not losing sight of the latter) and in ways that solidify and expand the current disciplinary repertoire. These science investments are needed to better define and develop the scope and range of social science engagement and the field of practice, build evidence of value-claims, standardize methods and tools, and push disciplinary boundaries. Figure 5 provides an overview of the six subdomains that emerged from our analysis and the 16 particular recommendations – all of which we discuss below in turn.

#### Agenda setting

Despite much enthusiasm, there remains uncertainty in what types of social science data, insights, and research should be prioritized at different stages of a response, or the location of entry points for social science integration across response activities (including, for example, clinical care, vaccination, epidemiology, water and sanitation, risk communication, community engagement, project management, logistics, security, administration and finance) and into the decision-making process. Models and budgetary options for integration have not been explicitly defined and explored in any systematic way that would allow for comparison and serious discussion. There is an important debate to be had about what is included and excluded by the term “social science” within the epidemic space, and how terminology will shape the field going forward. The lack of a shared language creates unnecessary obfuscations. As one key informants noted:

*“We understand that social science is important, and we know generally the questions and focus. But how to link research with operations, and embed it into a response framework, is the major unanswered question.” (Key informant, UN agency)*

Our respondents (many of which were directly involved with the West African 2014–16 Ebola epidemic) noted that by and large, medical anthropology has driven the momentum for change in preparedness and response structures in recent years. Yet, to build an inherently interdisciplinary and applied discipline greater inclusion of economics, political science, psychology, international relations, sociology, geography, history, and other disciplines is needed (see again Fig. 1 above). Many respondents from diverse institutional backgrounds expressed this. Some of these disciplines may not be relevant to the immediate needs of a response but have important contributions to make regarding the governance of health systems and medical humanitarian interventions, social determinants of health, and health policy. The goals, contributions, and character of this entire applied interdisciplinary field have not been well articulated, nor are the core competencies required to effectively integrate social science within the existing architecture.

Improving this situation will require a strategic and systematic process of agenda setting with core partners and allied disciplines. Particular recommendations that emerged from our analysis included: 1) generate consensus on top research and capacity building priorities; 2) create models for how to best embed social science into a response and existing structures and generate guidance for how agencies can build this capacity internally; 3) promote cross-disciplinary conversations to better delineate how different social science disciplines can contribute; and 4) convene a high-level expert group to identify barriers to social science involvement in the IHRs, and provide guidance to WHO on how to address them.

#### Core competencies training

Our results also showed that social scientists lack exposure to epidemic training and biomedical concepts, including the basics of epidemiology, emergency and policy frameworks, and the financing, ethics, and exigencies of the humanitarian system. As one informant noted:

*“Social scientists need to know the emergency response jargon, and key epidemiological terms. They need to understand WHO bureaucracy, and the institutional relationships between the big agencies, what the UN agencies do, also IHR and about the SDGs.” (Key informant, INGO)*

The core competencies needed to generate robust insights from time-pressured studies that accept uncertainty and (like outbreak epidemiology) generate rapid analysis and insights, have not been sufficiently developed. However, cadres of practitioner-scholars are needed who can conduct research as well as play key translational roles (as brokers, translators, and facilitators) and achieving this in practical terms will require field-based core competency training.

*“We need more people to be involved. We need to enlarge the community of practice, while at the same time having a really strong and dedicated core team.” (Key informant, social scientist)*

It is therefore necessary to better define core competencies and if possible institutionalize a field-training program (such as the WHO-led SocialNet initiative, and based on existing field epidemiology training programs, FETPs) including certification, simulation exercises, field learning, and training on basics of outbreak response. In addition, we need to develop curriculums and accepted training norms for core competencies and support key organizations in these training capacities over time.

#### Field-ready methodological tools

*“The time lag for real-time data to global level is a big problem. Everyone collects data differently and it is hard to aggregate. Really hard to enforce standards, and high staff turnover means people are in and out all the time.” (Key informant, funder)*

A third sub-domain in strengthening the “science” of social science includes basic investments to collate, standardize, test, and refine existing knowledge tools to make them fit-for-purpose. This was widely emphasized in our data. Knowledge, Attitude and Practice (KAP) studies continue to be the default or standard method, although the quality of existing studies and their appropriateness and relevance has long been of major concern [36]. Rapid qualitative assessments, community feedback, media analysis and community-led data collection systems have gained traction, but issues of quality, standards, best practice, metrics, and methods remain ill-defined. From a technology standpoint, the field is also lagging behind in using tablets for data collection and building databases for complex analysis that can integrate social variables. Much social science data gathering and analysis is still done with pen and paper (which is nonetheless the most appropriate strategy in many circumstances). Qualitative analysis software is proprietary, expensive, and difficult to learn. It is also unclear how to address the more personal aspects of anthropological fieldwork, including participant observation and field notes in remote analysis and the implications of this moving forward.

*“Social science knowledge needs to be "fit for purpose". There are rules, operational norms and standards that each organization has and it needs to fit within that architecture.” (Key informant, INGO)*

A number of important recommendations emerged from our analysis related to these issues. This included: 1) developing comprehensive fit-for-purpose handbooks for rapid social science methods in epidemics, for use by response agencies and applied field teams during different stages of an epidemic (which, surprisingly, do not exist); 2) creating and refining pre-positioned research protocols, which would involve developing a toolbox of methods and SOPs for research at critical integration points^1^; 3) supporting the creation of minimum standards, guidance, and tools to enable social scientists to work with response partners in developing community-led and responder/health system-led data collection systems that can collect integrated social science data, including rapid assessment methodologies, citizen social science, community-based rapid ethnography, and routine monitoring and evaluation data; and 4) supporting new, innovative streamlined data collection systems and technologies (tablets, apps, open access software).

#### Interdisciplinary research

*“In the epi [demiological] modelling space, we are not sure where social science can fit and how to engage with social science. We think maybe to have social science inform our baseline assumptions about a context, including the context of the response and our data. Also to help us understand the drivers for behaviors. This can include details about population density, for example. So we can communicate context, assumptions and uncertainties.” (Key informant, non-social scientist)*

While field-ready tools are an urgent necessity, so too are the application of other forms of interdisciplinary methods as well as novel, critical theoretical perspectives that expand current disciplinary boundaries. For example, risk communication and behaviour theories used in epidemic response are outdated and in need of re-assessment, although they continue to dominate response strategies and research. Furthermore, epidemiological data is difficult for social scientists to access, and the burden of constructing and maintaining large datasets, including personal health information, to conduct analysis linking social, economic and epidemiological variables and issues is a major roadblock to building the evidence-base. There is little investment in building integrated databases. Greater clarity is needed on how social science can and should be integrated with allied disciplines such as epidemiology, epidemic modelling, geospatial mapping, ecology, entomology, veterinary science, and big data and social analytics. All of this is required to push the boundaries of the current science and develop new, interdisciplinary science.

Five specific sub-domains emerged from our analysis. This include the need for: 1) strategic investments to support the epidemiology-anthropology tandem, or the strategic integration of social science data analytics into epidemiological datasets and networks, including better defining of opportunities and barriers to integration and data sharing; 2) better integration of social science in One Health preparedness and epidemic response, including real-time entomological, ecological, and zoonotic disease research and antimicrobial resistance; 3) developing methodological innovations focused on power, social networks and community resilience (especially important given the increasing interface between civil conflict and epidemics); 4) support for the translation of theory-driven research into practice, with specific focus on risk communication, community engagement and governance research; and 5) joint initiatives for projects with technology companies (i.e. Facebook, Amazon, Alibaba, Google or start-ups) interested in open source, ethical and/or community-owned data systems and analytics.

#### Building the evidence-base

*“The biggest challenge for social science to be included in the humanitarian sector is validation. Because it’s very hard to prove how social science insights influence program outcomes.” (Key informant, Social scientist)*

As noted in the introduction, there continues to be a widely acknowledged “evidence-gap” in how social science can improve epidemic preparedness and response, justifying reticence for some. There are, for example, few robust examples or case studies of social science improving a response, with most literature focused on Ebola [11, 12], but even here causative mechanisms are unclear. It is hard to quantify or measure the impact of qualitative studies, and more challenging to account for the cumulate effects of small day-to-day operational changes brought about by insights and attitudinal shifts by response managers and field teams as they approach and solve problems. While there are legitimate questions about demonstrating value (where, when, how, why, how much), without substantial investments in the field, value claims will remain anecdotal and suggestive. At the same time, supporting epidemic social science research should also be viewed as an essential part of promoting implementation science and operational research more generally, in order to evaluate the effectiveness of response strategies.

Specifically, there is a need to enable more operational social science research during and just after epidemics, focused on high-impact research gaps and questions about intervention effectiveness and response culture. This should include integrating social science methodologies into randomized controlled trials, observational studies and longitudinal data collection that tracks effectiveness over time. In addition, there is a need in long-term support for process evaluations and the documentation of success stories and lessons learnt to promote reflexive learning, as well as a need for large-scale mixed methods studies in high-priority countries to explore preparedness issues.

#### Ethical issues

*“We need a code of conduct for social science in outbreaks, of how to do respectful and meaningful research that does not burden the population, especially with patients and their families.” (Key informant, social scientist)*

A final part of basic science strengthening involves ethical and moral reflection and the need to develop guidelines and codes of conduct. Our data supports the need for concerted work on epidemic response ethical standards and the conflict between institutional priorities and the needs and wants of communities, including during periods of recovery, as well as codes of conduct and legal recourse in instances of abuse and unethical behaviour. There are also disciplinary ethical questions that need to be engaged, including the effects of research on the response and recourse to unintended consequences. This includes multiple researchers interviewing the same affected patient or family and creating research fatigue, for example.

Some particular recommendations included: 1) convening research on the ethical dimensions and dilemmas of social science research and applied fieldwork and applied fieldwork in epidemic preparedness and response, including how research can be meaningfully accountable to communities; and 2) supporting the development of a social science code of conduct in epidemics, including legal and institutional guidelines (and compensation in case of researchers being injured, sick or killed), in coordination with ethicists and by looking to other disciplines in the epidemic response space (clinical trials and humanitarian ethics, for example).

### A supportive social science ecosystem

**Fig. 6:**
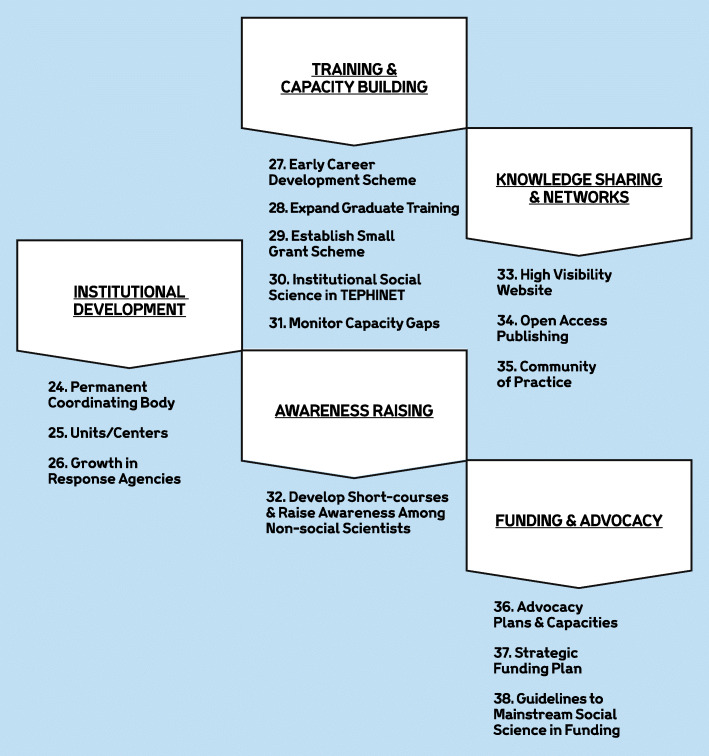
A supportive social science ecosystem

Our analysis found that a broader ecosystem of knowledge, infrastructure and funding (Area 3) is needed to support, in different ways and at different temporal scales, the development of core epidemic social science response capacities (Area 1) and the strengthening of basic science (Area 2). These foundational elements would facilitate the flow of resources and information, strengthen the growth of the broad range of competencies, capacities, and capabilities discussed above, and create durable resilience. Growth of the disciplinary ecosystem, − dependent on the five sub-domains outlined in Fig. 6 – will depend on how strong this supportive and foundational ecosystem becomes.

#### Institutional development

*“We need a permanent operational budget to engage in advocacy and long-term strategic planning, training, publications, learning and collaborations.” (Key informant, social scientist)*

*“There needs to be a unit set-up in each organization to help develop social science capacities. They need to implement this from the inside, shake things up from inside.” (Key informant, INGO)*

Our analysis found that the growth of agile social science research units and centers that actively engage in preparedness and response was seen among many respondents to be fundamental for knowledge generation and capacity strengthening. In the near-term, these should leverage existing epidemic response networks and institutes, for example embedded within public health agencies, humanitarian organizations or reputable biomedical research centers. At the time of our research, a great deal of work rested on the shoulders of a few key innovators, without much institutional infrastructure, focused around specific diseases. Projects are funded short-term without mechanisms for collaboration, onboarding strategies to grow teams, or the means to rapidly deploy and coordinate field activities, or re-orientate resources to prepare for and respond to new epidemics. There are systemic deficiencies in research infrastructures in middle and low-income countries that need to be addressed – capacities that are often taken-for-granted in northern institutions.

*“It is hard to move money from universities in North to South, even for major biomedical studies from prestigious universities. It can take months! Researchers have to pay out of pocket and get reimbursed (sometimes not even). Even flagship clinical trial projects are like this.” (Key informant, social scientist)*

In the global south, lack of administrative and office staff, project managers and data management capacity are major impediments to research, but so too are a lack of basic internet, journal access, and grant writing capacity. Academic partnerships often do not address these systemic administrative capacity gaps. Epidemic social science, therefore, needs a program of institutional development in order to ensure appropriate growth, advocacy, and communication capacity to bring its full expertise to the table. Without this, insights cannot be engaged at key policy, resource mobilization, agenda setting, and prioritization points in epidemic management decision-making.

Our results highlighted the need for three significant recommendations in institutional strengthening: 1) a need to establish a permanent non-profit coordinating body for advocacy, administration, and capacity building, like a common service platform or secretariat, with permanent staff and a multi-country presence (It is important to note that not all respondents agreed with this particular recommendation, as there were concerns that this would “centralize” the field in the hands of northern partners if not designed in an appropriate manner); 2) the development of a global network of fit-for-purpose epidemic social science units and centers, especially in crises-prone countries in the global south, to function as innovation accelerators and WHO collaborative centres (and supported with a package of institutional and infrastructural support); and 3) medium and long-term growth of social science capacity in response organizations and in national preparedness and response plans.

#### Training and capacity building

*“There needs to be social science across the whole organization, in all trainings, all needs to be touched by social sciences. There needs to be social science for dummies trainings to make all pillars aware of the value and relevance” (Key informant, INGO)*

As already noted, there is a need to increase the competence of social scientists in basic epidemiological and public health skills and in emergency national and international systems and frameworks in order to facilitate their relevance and capabilities. In the global south in particular, there is a need to invest in long-term capacity building to address systemic barriers: lack of grant writing capacity, mentorship opportunities, publication incentives, and English language skills (the lingua franca of the international system); these issues were particularly emphasized by key informants based in the global south.

A number of important recommendations are worth emphasizing in this sub-domain. The first is the creation of an early career development fellowship scheme that would tie together national funding for medicine, public health, and social sciences into a dedicated career track is a strategy to improve training. A set of priority countries could be selected to pilot such a scheme. In addition, graduate and post-graduate training and education on social science, infectious disease and epidemics should be expanded along the continuum of applied-academic research, in the basics of outbreak response and in ways that facilitate opportunities for field-based learning, internships, exchange programs, and career development. A small grants (seed-fund) scheme should be created for researchers from the global south, as a way to support innovation and jump-start capacities (e.g. modelled from the Special Programme for Research and Training on Tropical Disease’s (TDR) small grant initiatives). Many social science capacities, in fact, can be institutionalized in existing epidemiology networks, notably TEPHINET and other key national Field Epidemiology Training Programs (FETPs). Also important here is the development of indicators to monitor epidemic social science capacity at country and organization level – for example, as part of the Global Health Security Agenda’s Joint External Evaluations (JEEs).

#### Awareness-raising with allied disciplines

The social science ecosystem also depends on allied disciplines to understand social science contributions and value and to be socialized in its requirements and norms. In one regard, this will demand changing negative perceptions that many biomedical researchers and response partners may have about social science: as irrelevant, vague, un-scientific, and too theoretical. This reticence is driven by a lack of integration and appreciation of social contexts in science education more generally, as well as limited exposure to qualitative mixed method and ethnographic data. This also extends to the receptivity of national Institutional Review Boards (IRBs), who need to be socialized in how to handle qualitative and community-led data collection and participatory methods. Lastly, there is also an urgent need to increase understanding among funders, governments and response agencies of the finance, capacity, and logistics needs of social science studies. There is a dominant perception that social science can be supported with a small, ad hoc pool of funds, and there is a lack of appreciation of the time and training requirements involved in data collection that needs to be changed. While epidemiological studies are regularly accepted to take months or longer to complete, all forms of social studies are expected to be completed within much shorter timeframes.

*“Government does not include social scientists in outbreak teams. The government is not aware of the value of qualitative social science and multidisciplinary teams. Whenever I go to meetings, I am the only social scientist, for many many years now. People are confused and ask me what anthropologists do; they confuse it with entomology and archeology.” (Key informant, social scientist)*

To address these systemic barriers, there is a need to develop and deploy awareness-raising and short training material for non-social scientists to become better acquainted with social science research. Training courses should be geared to everyone within an organization, including incident managers, human resources, security, and logistics professionals, and include materials and initiatives for national ethics committees to enable better social science review and approval. At the same time, these issues should also challenge social scientists to think critically about how they present themselves and their findings to allied disciplines so that they can grasp their value.

#### Knowledge sharing platforms and networks

*“There are not very many academics focused on knowledge translation in the operational research and epidemic space. In fact, it can be very challenging to publish operational studies, based on small sample of qualitative interviews. But, really, I have been surprised by how much impact articles can have. Practitioners are really interested in publishing their lessons and working with the academic community.” (Key informant, non-social scientist)*

Knowledge sharing platforms and networks are an important part of supporting the integration of epidemic social science and the growth of new knowledge and approaches. This includes a commitment to open access publishing, the centralization of resources on the web, an expert database, and various face-to-face opportunities for social scientists to attend conferences, organize and strategize, and share knowledge.

The support of a high-visibility website with multiple modalities would be a useful strategy. This could build on the existing Epidemic Response Anthropology Platform (ERAP) (https://www.epidemicresponse.net) or similar types of platforms. In addition, there is a need to continue and expand support for open access publishing, especially among practitioners and researchers from the global south. A further recommendation that emerged from our data related to the need to develop a community of practice including a long-term administration of an online database of epidemic social science experts and a professional association with an annual conference to support peer-to-peer learning.

#### Funding and advocacy

*“We need an advocacy group to sit at the table, to push organizations to have social science be part of the response before, during and after.” (Key informant, UN agency)*

Finally, funding was often emphasized an important part of growing the field of epidemic social science. The field was believed to benefit significantly from greater concerted advocacy and communication strategies and capacities to help create momentum, visibility, and generate additional partnerships. In addition, it is imperative that the community as a whole thinks both outside and inside the current funding box, finding ways to maximize resources and synergies with existing initiatives while also generating new forms of investment and support. There are a number of ways that funders can assist with creating momentum that emerged from our analysis. One is the development and deployment of advocacy plans and capacities, targeting different high priority stakeholders, to raise the profile and visibility of social sciences in the epidemics field. In line with this, support for a strategic plan to broaden the funding landscape is needed, including an annual Funders Forums that invites a broad range of stakeholders (the first such forum was organized by GloPID-R in Tokyo in 2019: https://www.glopid-r.org). In fact, guidelines and expectations for funders to mainstream epidemic social science in funding determinations could be an important contribution for sustained support. Funders could allocate a certain percentage of epidemic response funding to social science research and establish expectations that social science must be integrated into new or current high-impact projects and initiatives, and fund onboarding mechanisms for current initiatives that do not (i.e. CEPI).

## Discussion

Epidemic preparedness and response, as well as health systems strengthening initiatives are increasingly recognizing epidemics as complex biosocial events – epidemiologically, clinically, socially and geopolitically [37, 38]. Day-to-day decision-making – by communities, frontline health staff and humanitarian responders – takes place in a context of uncertainty, complexity, fear and stress across different temporal and spatial scales, embedded within the forces of politics, history and the unequal and inadequate distribution of resources.

Addressing public health emergencies requires, among other things, the effective use of knowledge and expertise. The rollout of response capacities and capabilities, from national and global to field-level pillars and community-led activities, are predicated upon the quality, course and timeliness of key information flows and knowledge synthesis. It is widely accepted that epidemiological data is essential for an effective epidemic response [39], as seen by the continued growth of the applied field of outbreak analytics [40]. However, this level of professionalization and integration has only limitedly been advanced for social science. Furthermore, different streams of data – for example, epidemiological and anthropological – are not currently integrated in any systematic and meaningful way [41]. A future *science of outbreaks* will need to consider how to rapidly integrate multiple sources of information [42].

Epidemic social science, as an emerging field of practice, is already generating new forms of operational data and insights to facilitate critical self-reflection and adaptive learning – for example, in the 2018–20 Ebola epidemic in eastern DRC [11, 12]. Among the 75 experts consulted for this paper, and based on additional focus groups and literature review, we found a general feeling that the widespread adoption of social science techniques, and better integration of community knowledge and participation, will challenge the status quo of the existing humanitarian system, scientific and medical education and global and national governance regimes. In this sense, social science knowledge may also represent a challenge to the institutional status quo, because its analysis may identify these institutions themselves (and the response architecture and ethos) as part of the problem. From this perspective, social science engagement needs to be viewed as an “essential activity” in order to overcome the vested interests and inertia in the scientific status quo.

Nevertheless, there is a need to manage expectations. Social science is sometimes seen as a tool –“the keys” – to “unlock” community acceptance. This narrow, instrumental view has precedence in the early role of social science in the HIV and TB fields in the 1990s, which conceptualized these (re) emerging diseases as behavioural problems that could be solved only by behavioural change techniques [20]. Within response agencies, social science has most frequently been siloed within the risk communication, health promotion and community engagement fields, to focus on community resistance and compliance. This is obviously an important area that should not be understated, but there is an urgent need to broaden and clarify the field’s value and contributions beyond this narrow remit. The COVID-19 pandemic has now substantially challenged this narrow vision. It must also be acknowledged, however, that greater professionalization of social sciences in epidemics, in and of itself, remains only a partial response to the deep political-economic and governance challenges – both in states and donor institutions – that prevent long-term structural change. Lastly, it is important that the field engages with current debates to “decolonize global health” and ensure greater and diverse leadership by practitioners based in the global south, especially crises-prone countries [43].

In this paper, we have outlined a framework for how epidemic response stakeholders, including funders, can help address core gaps that prevent social science integration in epidemics. This has included three overlapping areas: 1) core capacities, 2) applied and basic science, and 3) the growth of a supportive disciplinary ecosystem. The strategic recommendations that developed out of this exercise are not without precedence. If we look to the development of allied scientific disciplines that are now essential parts of the global epidemic response architecture, we find historical antecedents for the professionalization process. This includes virology in the early 1900s and, especially, field epidemiology in the 1970s/80s ([44–47]. These disciplines underwent substantial periods of sustained core capacity building, growth in the applied and basic science continuum, and broad global and national investments in institutionalization. Further exploration of the relevance of this disciplinary history to the applied field of epidemic social science should be pursued. While early advances have been made in the field of epidemic social science, these need to be leveraged and expanded upon, supported with a similar level of investment today that these allied disciplines received in the past.

This research was conducted just prior to the 2020 COVID-19 pandemic. In this sense, it represents an analysis of the institutional landscape right before this global pandemic. Future analysis could build upon our framework to explore what has changed, or not changed, due to COVID-19. Will we look back and see the 2020s as the core period of growth in the field of epidemic social science?

## Conclusion

Over the last few decades, interest in *how* social science can contribute to epidemic and pandemic control has increased. In this paper, we have presented a framework that outlines a range of investments and opportunities to strengthen social science integration. Our analysis was conducted and written before the COVID-19 pandemic, which reminded us, once again, of the social, cultural, political and economic contexts and consequences of infectious disease. Now is the time for social scientists, funders, global agencies, allied disciplines, and national governments to strategically build core capacities and competencies, and move epidemic social science from the margins to the mainstream. In so doing, social science will challenge the existing status quo. We believe this will make it more people-centric and responsive to the needs and challenges of diverse human communities in the twenty-first century – a century that is widely predicted to witness many more disruptive public health emergencies.

## Data Availability

N/A

## Abbreviations

CIHR: Canadian institute of health research
ERAP: Epidemic response anthropology platform
GOARN: Global outbreak alert and response network
KAP: Knowledge, attitude and practice
RCCE: Risk communication and community engagement
SSHAP: Social science and humanitarian action platform
UK-PHRST: UK-public health rapid support team

## Footnotes

1 This could include 1) transmission and spread; 2) case surveillance; 3) disease emergence and mitigation at animal-environmental source; 4) evaluating interventions; 5) RCCE; 6) local health care; 7) recovery; 8) clinical research; and 9) preparedness.
